# The Nursing Student Licensure Examination: A Scoping Review

**DOI:** 10.3390/nursrep15080299

**Published:** 2025-08-14

**Authors:** Flavia Pantaleo, Alessandro Stievano, Chiara Mastroianni, Giorgia Petrucci, Natascia Mazzitelli, Michela Piredda, Maria Grazia De Marinis, Anna Marchetti

**Affiliations:** 1 Department of Biomedicine and Prevention, University of Rome Tor Vergata, 00133 Rome, Italy; 2 Department of Clinical and Experimental Medicine, University of Messina, 98122 Messina, Italy; alessandro.stievano@gmail.com; 3 Department of Life Science, Health, and Health Professions, Link Campus University, Casale di San Pio V ST, 44, 00165 Rome, Italy; chiara.mastroianni@gmail.com; 4 Research Unit of Orthopaedic and Trauma Surgery, Campus Bio-Medico University Hospital Foundation, Alvaro del Portillo ST, 200, 00128 Rome, Italy; g.petrucci@policlinicocampus.it; 5 Centre of Excellence for Nursing Scholarship OPI of Rome, Degli Ammiragli ST, 67, 00136 Rome, Italy; natasciamazzitelli@gmail.com; 6 Department of Medicine and Surgery, Research Unit Nursing Science, University Campus Bio-Medico, Alvaro del Portillo ST, 200, 00128 Rome, Italy; m.demarinis@policlinicocampus.it (M.G.D.M.); a.marchetti@policlinicocampus.it (A.M.)

**Keywords:** undergraduate nursing, final exam, licensure exam, nursing licensure, nursing student

## Abstract

**Background**: In an increasingly globalized context marked by growing professional mobility, establishing shared standards for assessing nursing competencies is essential. The licensure examination represents a critical gateway between academic preparation and professional practice. However, significant ambiguity remains regarding what competencies are assessed and how this assessment is conducted internationally. **Objective**: This scoping review aimed to map the international literature on nursing licensure examinations by comparing frameworks and domains, performance levels, and assessment tools to identify similarities and differences in the core competencies required for entry into practice. **Methods**: The review followed Arksey and O’Malley’s methodological framework. Comprehensive searches were conducted across PubMed, CINAHL, Scopus, ERIC, Cochrane Library, ProQuest, and OpenGrey databases. Studies addressing competency frameworks, performance levels, and assessment tools in undergraduate nursing licensure were included. **Results**: Twenty-three studies were analyzed. The most frequently cited framework was ‘Client Needs’. Competency domains commonly addressed patient needs, professional roles, and healthcare settings. The dominant performance level was cognitive, typically assessed through multiple-choice questions; practical skills were often evaluated via ‘bedside tests’. Despite variations in frameworks and domains, cognitive performance expectations and assessment tools showed some consistency. **Conclusions**: These findings underscore the need for a context-sensitive, internationally adaptable framework to promote fairness and support nurse mobility.

## 1. Introduction

In recent years, globalization has significantly increased the international mobility of workers and professionals. Among them, nurses are increasingly practicing in countries other than their own [1].

Furthermore, the increasing complexity of healthcare systems, multimorbidity, and the growing prevalence of chronic and acute conditions have further intensified the need to train highly competent nursing professionals capable of managing diverse and ever-changing clinical scenarios [2].

At the same time, global population aging has heightened the demand for qualified nurses, thereby incentivizing international mobility and underscoring the urgency of investing in nursing education [1]. The global shortage of nursing staff has long been recognized as a critical issue. Countries such as the United States and the United Kingdom have, for years, relied on recruiting qualified nurses from abroad to address domestic workforce gaps [3]. Although projections suggest that this shortage could decrease to 7.6 million by 2030 [4], it remains a persistent structural challenge that threatens the sustainability of healthcare systems.

In this increasingly interconnected healthcare landscape, establishing internationally shared competency standards is essential. Such standards enable nurses to operate effectively across different geographical settings, promoting health and protecting patients irrespective of national boundaries [1]. These standards typically define the minimum performance thresholds required for entry into clinical practice [1]. However, a barrier to harmonizing competency assessment lies in the lack of a universally accepted definition of competence. This challenge is compounded by the complexity of certification processes at the end of educational pathways [3].

The concept of competence has been a central topic in international discourse across various professions, including nursing, and has been interpreted differently depending on disciplinary and cultural contexts [5]. Garside and Nhemachena [6] describe it as an entity that is difficult to describe in concrete terms, while Cassidy [7] defines it as a combination of knowledge, skills, and attitudes. A more comprehensive perspective is proposed by Meretoja et al. [8], who define competence as the functional capacity to integrate knowledge, skills, attitudes, and values in specific care settings.

In most contexts, competency certification is formalized through a final licensure examination, which serves as the official mechanism for assessing the readiness of nursing graduates and authorizing their entry into the profession [9]. However, international regulatory frameworks differ widely in how licensure examinations are implemented. Some countries have long-established systems, while in others, regulatory processes are still evolving [10]. For example, the United States, China, Japan, and Thailand require a national licensure examination [11]. In contrast, in Spain, completion of a university degree allows direct registration with the professional board, without further testing [10]. A similar model applies in England, where academic qualifications permit registration with the Nursing and Midwifery Council (NMC) without a final examination [10]. In Italy, however, a licensure examination is mandatory to formally certify both theoretical and practical competencies [10,11].

In this review, the term “nursing licensure examination” refers to an examination administered by governmental or professional regulatory bodies, typically at the national or state level, which authorizes graduates of accredited nursing programs to enter professional practice. These examinations are distinct from academic or university-level assessments and are usually required regardless of the institution where the nursing degree was obtained. The structure, content, and regulatory framework of licensure examinations vary internationally, reflecting differences in educational systems and professional standards [10].

Regardless of the specific methods used, all systems share the expectation that, upon completing their education, nursing students must demonstrate the competencies necessary to address priority health problems and deliver safe, effective care [1,2]. Several studies have highlighted significant variation not only between countries, but also within individual national contexts regarding the competencies considered ‘core’ for new graduates [11,12,13]. To address these discrepancies, academic and professional community have worked to develop competency frameworks, domains, and performance levels conceptualized as expected learning outcomes [14].

In the United States, for example, the American Association of Colleges of Nursing (AACN) published The Essentials: Core Competencies for Professional Nursing Education (2021), which outlines a comprehensive framework for competency-based nursing curricula and serves as a national standard for CCNE-accredited institutions and reflects a growing emphasis on harmonizing education with professional expectations and licensure requirements [15].

In the European context, these levels generally refer to the Dublin Descriptors [16], which include knowledge and understanding, applying knowledge and understanding, making judgements, communication skills, and learning skills.

In the European context, the Bologna Process represented a significant effort to harmonize education systems across countries; the process was operationalized through the Tuning Educational Structures in Europe project [17]. Within the field of nursing, this initiative led to the development of two key competency frameworks: the Tuning Nursing Specific Competences [18], which outlines 47 competencies grouped into six domains, and the EFN Competency Framework [19], which is also organized into six domains. In parallel, several studies have described the tools used for the evaluation of competencies during the licensure examination. These tools include open- or closed-ended response tests, clinical cases discussions, bedside practical assessments, procedural protocols, and structured evaluations such as the objective structured clinical examination (OSCE) [10,11,12,13]. However, preliminary bibliographic searches conducted in major databases including PubMed, Cochrane, JBI, and PROSPERO revealed a limited number of studies that examine in detail what is assessed and how the assessment is conducted during the nursing licensure examination [13,20,21].

## 2. Materials and Methods

### 2.1. Objective

The objectives of this study were as follows:

1. To map the international literature on the nursing licensure examination;
2. To compare the frameworks and related domains of the competencies assessed the performance levels, and the tools used for their assessment, to highlight convergences and divergences in the core competencies assessed in the licensure examination.

### 2.2. Study Design

The scoping review represents a methodological approach useful for mapping and integrating the available evidence on a given phenomenon.

This study adopted scoping review design, which is particularly appropriate for topics that are complex or heterogeneous and have not been extensively reviewed. The scoping review approach was deemed suitable to map the breadth and depth of the existing international literature on nursing licensure examinations, which vary in purpose, structure, and implementation across countries. Unlike systematic reviews, scoping reviews do not aim to critically appraise individual studies but rather to identify and categorize existing evidence, highlight knowledge gaps, and provide an overview of key concepts and approaches.

This study was conducted in accordance with the methodological framework proposed by Arksey and O’Malley [22], which is divided into five stages: (1) the definition of the research question; (2) the identification of relevant studies; (3) the selection of studies; (4) data collection and organization; and (5) the synthesis and presentation of results.

While Arksey and O’Malley’s scoping review framework provides a robust methodological foundation for mapping existing literature, it does not inherently include a theoretical lens for interpreting the phenomena under study.

To enhance the conceptual understanding of nursing licensure examinations, this review acknowledges the potential relevance of established theoretical models such as the job demand–resources (JD-R) model [23], which explores how job demands and available resources impact individual well-being and performance. Additionally, the conservation of resources (COR) theory [24] offers insight into how new nursing graduates manage personal and professional resources during stressful transitions, such as licensure exams. Trauma Theory [25] may also provide a valuable framework for understanding the emotional challenges faced by internationally mobile nurses navigating unfamiliar certification processes.

No protocol has been registered with the Open Science Framework.

### 2.3. Stage 1, Identifying the Research Question

To answer the objective of the review, the following research questions were formulated: What competency framework is used for assessment during the nursing licensure examination? What are the expected performance levels for passing the licensure examination? Which tools are used for the evaluation of competencies?

In this review, ‘nursing licensure examination’ refers to the final examination at the bachelor’s degree level, which clarifies the transition from student status to that of a licensed professional.

### 2.4. Stage 2, Identifying Relevant Studies

In December 2022, preliminary searches were conducted in PubMed, Cochrane Database of Systematic Reviews, JBI’s Evidence-based Practice Database (Ovid) and PROSPERO that confirmed the absence of other protocols or of scoping reviews already published on the topic.

The databases consulted for this review were: PubMed, CINAHL (EBSCO), Scopus (Elsevier), ERIC, and Cochrane Library. The gray literature search included ProQuest Dissertations and Theses and the OpenGrey portal (www.opengrey.eu/). Studies published between 1 January 2000, and 1 December 2024, in English and with an abstract, were included. The selected time interval is justified by the launch, in 1999, of the Bologna Process, a European intergovernmental agreement aimed at reforming higher education through the introduction of a system based on three comparable educational cycles [16].

The Bologna Process also represented an important impetus to the harmonization of educational standards at the international level, potentially influencing the emergence of new models of licensure examination.

The keywords (thesaurus terms and text words) appropriate for the research questions, were combined using the Boolean operators AND, OR, or NOT and adapted to each database. The full texts of the selected articles were searched manually to identify further relevant sources (Appendix A.1).

International peer-reviewed studies with results pertaining to frameworks and domains of competence, performance levels, and competency assessment tools used during the licensure examination were included. The review included: quantitative studies (experimental, quasi-experimental, analytical, and descriptive observational), systematic or scoping reviews, qualitative studies, expert opinions, discussion articles and the gray literature (e.g., technical reports, summaries). Studies referring to licensure examinations of health professions other than nursing; articles published before 2000 or not in English; reports, conference proceedings, research protocols, and posters were excluded.

### 2.5. Stage 3, Study Selection

The search yielded 3446 records, which were subsequently imported into EndNote Clarivate Analytics v.14.0. Following the removal of duplicates (n= 606), two reviewers (F.P. and A.S.) screened the titles and abstracts. After a pilot test conducted on 23 studies, the reviewers proceeded with independent screening. Full texts of articles deemed potentially relevant were retrieved for further evaluation. In instances of uncertainty or disagreement, a third reviewer (A.M.) was consulted to reach consensus. The selection process followed the scoping review checklist [26].

The PRISMA-ScR is a 27-item checklist designed to improve the clarity, completeness, and transparency of scoping reviews, particularly those addressing complex or diverse research areas [27].

In the planning and reporting phases of this review, the checklist was used to ensure that key components, including the review rationale, detailed eligibility criteria, comprehensive search strategy, selection process, data representation methods, and summary of findings, were explicitly described. Use of the PRISMA-ScR also supported a structured presentation of findings and enabled better alignment with the methodological standards recommended by the Joanna Briggs Institute.

A complete PRISMA-ScR checklist is included in the Supplementary Materials to ensure transparency and reproducibility (see Supplementary Table S1) and the results were reported in accordance with the PRISMA-ScR flow diagram [26].

### 2.6. Stage 4, Data Charting

Data extraction was conducted using a systematic approach [22]. Two independent reviewers (F.P. and A.S.) employed a purpose-built extraction tool (Appendix A.2), which included the following elements: study characteristics, competency frameworks and domains, performance levels, and assessment tools. The tool was refined throughout the extraction process, with all modifications shared among the research team. When critical information was missing, the authors of the included studies were contacted to request its provision.

### 2.7. Stage 5, Collating and Summarizing

All authors agreed on the method of presenting the review data following a thorough, collaborative analysis of the results. The methodological quality of the studies included was initially assessed independently by two reviewers (F.P. and A.S.), using the tools developed by the Joanna Briggs Institute [28]. (J). Agreement was achieved through direct comparison and the use of an evaluation grid comprising four response options: “yes,” “no,” “unclear,” and “not applicable.”

For each of the three research questions addressed in the scoping review, the extracted data—pertaining to the type and purpose of the studies, as well as key findings—were organized and summarized in tabular form. This included information on the competency frameworks and domains assessed during the licensure examination, the expected performance levels, and the assessment tools employed.

Narrative synthesis accompanied the tabular data, highlighting similarities and differences among the studies for each variable considered. This approach aimed at providing a comprehensive and comparative overview of the practices adopted across various international contexts.

## 3. Results

### 3.1. General Description of the Studies

**Figure 1:**
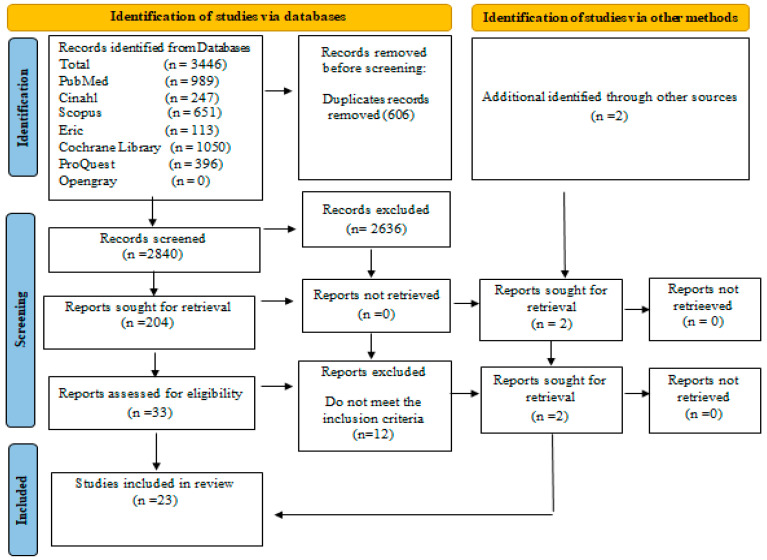
PRISMA diagram of literature search [26].

Twenty-three records were included in this review (Figure 1), comprising twenty original research articles, two doctoral dissertations, and one expert opinion, all published between 1 January 2000, and 1 December 2024.

**Figure 2:**
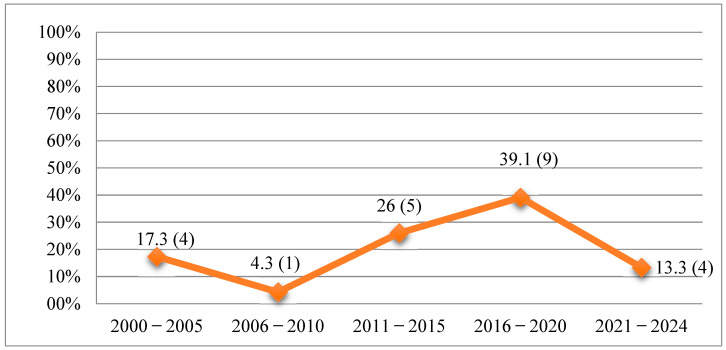
Percentage and number of articles published by year/class (N = 23).

**Figure 3:**
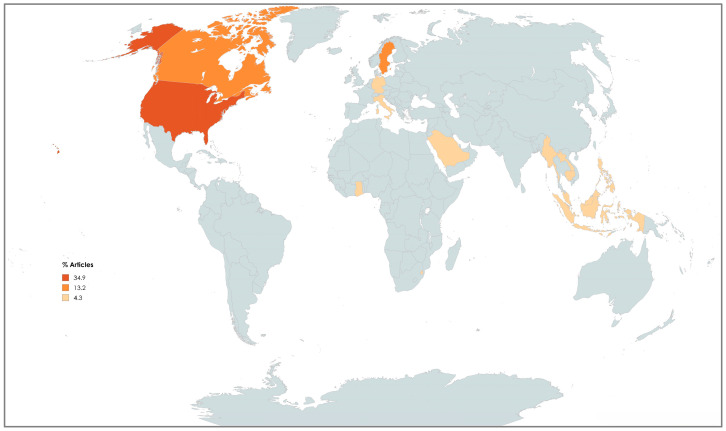
Choropleth of document distribution by geographic region of studies that have been conducted in following countries: Canada 13.2; United States of America 34.9; Commonwealth Caribbean 4.3; Ghana 4.3; Eswatini 4.3; ASEAN 4.3; Korea 4.3; Indonesia 4.3; Saudi Arabia 4.3; Germany 4.3, Italy 4.3; Sweden 13.2.

**Figure 4:**
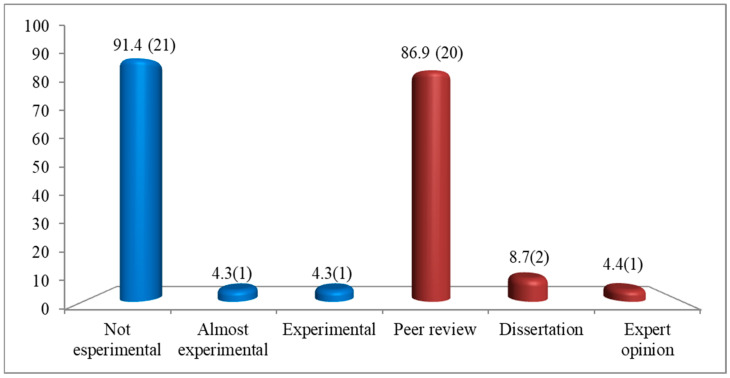
The number and percentage of studies categorized by type in the scoping review (N = 23). Legend: blue color columns = study design; red color columns = type of study.

Most selected records were published during the five-year period from 2016 to 2020 (n = 9, 39.1%) as shown in Figure 2. Most studies were conducted in America (n = 12), followed by Europe (n = 5), Asia (n = 4), and Africa (n = 2) (Figure 3). Further details regarding the characteristics of the included studies are presented in Figure 4 and Table 1.

### 3.2. Framework Competencies Assessed in the Nursing Licensure

The objective of this review was to map the international literature on nursing licensure examinations, with a focus on competency frameworks and domains, expected performance levels, and assessment tools. Data analysis identified 13 competency frameworks described in 21 of the 23 included articles (Table 2).

In one of these studies, the framework was presented without specifying its competency domains [45] while another study reported only the domains [44], and one study did not include either a framework or domains [36] (Table 2).

Regarding performance levels, five theoretical reference models were identified. Eleven studies explicitly referenced these frameworks, while eight studies reported performance levels without indicating a theoretical basis [29,34,36,40,41,43,44,45] (Table 3).

All 23 studies included in the review reported on the assessment tools used in nursing licensure examinations. These tools can be categorized into six types for cognitive testing and three for practical evaluation (Table 4).

In response to the first review question—"What competency framework is used for assessment during the nursing licensure examination?”—a total of 13 distinct competency frameworks were identified and are detailed in Table 2. These frameworks were described in 21 out of the 23 included studies. The most frequently cited was the Client Needs framework, which appeared in nine studies—eight conducted in the United States [33,35,41,43,46,47,48,49] and one in Canada [40]. This framework is structured into four domains—safe care environment, health promotion, disease prevention, and health promotion—and emphasizes the identification and fulfillment of patient needs across various care settings.

Other competency frameworks emerged across the included studies, each reflecting the specific context of the country or region in which it is applied. For example, the SNLE Guideline [29], adopted in Saudi Arabia, includes four domains; the NMC Standards [30], used in Ghana, consist of six domains; and the SoS Swedish Declaration [31], implemented in Sweden, is composed of four domains. In Canada, the CASN framework [32,51], identifies six domains, while in the ASEAN region, the AJCCN framework encompasses five domains shared among ten member states.

In addition, the QSEN framework [21], also adopted in Sweden, is structured into six domains; the Tuning Nursing Specific Competences [38], used in Italy, includes five domains; and the ENC Framework [37], applied in Eswatini, is composed of seven domains with a focus on person-centered care. The ETPC [39], used in Canada, the competencies grouped into six domains, while the CARICOM Blueprint [42], is derived from the curricula of 13 nursing schools across the Caribbean. The AINEC minimum standards [45], implemented in Indonesia, does not define specific domains. Finally, the Angoff method [50], used in South Korea, establishes minimum competency thresholds through eight domains as part of the performance level description (PLD).

The predominance of the Client Needs framework in the included studies can be attributed to the high number of U.S.-based publications, many of which are focused on predicting students’ academic and professional success through outcome measures such as exit exams, GPA, and NCLEX-RN scores [33,35,40,41,43,46,49].

Regarding the second research question—"What are the expected performance levels for passing the licensure examination?”—five theoretical frameworks used to define performance expectations in nursing licensure examinations were identified, as detailed in Table 3. In the table, studies were classified as “not specified” when there was no explicit reference to any performance level framework, such as Bloom’s taxonomy [52], Miller’s pyramid [53], or similar hierarchical models. In these cases, it was not possible to infer a performance level due to the absence of detailed descriptors or structured classification criteria provided by the authors.

Among the frameworks identified, Bloom’s taxonomy was the most frequently cited, being adopted in five studies [33,46,47,48,49]. The revised version of Bloom’s taxonomy by Anderson and Krathwohl [54] organizes cognitive skills into six hierarchical levels: Remember, Understand, Apply, Analyze, Evaluate, and Create.

Miller’s pyramid was employed in three studies [21,31,32], providing a model of clinical competence that progresses through four stages: Knows, Knows How, Shows How, and Does [53].

The national clinical judgment measurement model (NCJMM) appeared in one study [35]. This model, which is grounded in Tanner’s Clinical Judgment Model [55]. outlines four levels of clinical reasoning.

The Dublin descriptors, used in one Italian study [38], define five levels of expected learning outcomes at the conclusion of a degree program, as established by the Italian Ministry of Education, University and Research [56].

Lastly, the Standard Clinical Competencies Record Book (SCCRB) serves as the official framework for performance assessment in Eswatini [37].

The analysis highlights the predominance of Bloom’s taxonomy, particularly in U.S.-based studies, where the emphasis on cognitive development—especially within the domains of knowledge and clinical judgment—is viewed as a critical foundation for professional readiness [33,35,41,43,46,49].

Regarding the third research question—“Which tools are used for the assessment of competencies?”—two main categories of assessment emerged—cognitive and practical—as detailed in Table 4.

Cognitive assessment was reported in all 23 included studies and was most frequently conducted through written examinations (Table 4).

Additionally, some studies reported the use of oral examinations as part of the cognitive assessment [38,44].

Six distinct types of cognitive assessment tools were identified across the studies. Multiple-choice questions (MCQs) were the most commonly employed, appearing in 17 studies either in paper-based or computerized formats. Modified essay questions (MEQs), which involve clinical scenarios with open-ended questions, were reported in two studies. A hybrid format combining MCQs and MEQs was found in three studies. Open-ended response tests, focused on recall and argumentation, were reported in two studies. Clinical case resolution, either real or simulated and presented in written or oral formats with an emphasis on problem-solving, was described in five studies. Finally, discussion of protocols and procedures was used in two studies as part of competency evaluation (Table 4).

Overall, MCQs represented the most frequently used cognitive assessment tool, followed by clinical case resolution. Notably, only one study did not specify the type of written examination employed [44].

Practical assessments were classified into three main modalities. Simulated clinical cases (OSCEs) were described in two studies, where standardized stations were used to evaluate clinical performance [30,38]. Bedside tests, applied in five studies, evaluated student performance directly in clinical settings either individually or in teams [21,31,36,38,44]. Lastly, decontextualized practical tests, focused on isolated technical skills, were reported in one study [38].

Among the studies that included practical testing, bedside tests were the most commonly used, followed by OSCEs. However, 17 studies did not report any form of practical assessment. Notably, only one study [44] incorporated all three types of assessments: written, oral, and practical.

## 4. Discussion

The objective of this scoping review was to map the international literature on nursing licensure examinations, comparing the frameworks and domains of assessed competencies, the expected performance levels, and the tools used in assessment.

The analysis revealed considerable heterogeneity in the reference frameworks used across studies. Competency domains are structured according to different and often non-overlapping categorical systems, which complicate the establishment of standardized international benchmarks. In general, the frameworks reference three primary areas: patient needs, nursing roles/functions, and areas of care [32,33,34,35,37]. As noted by Oermann et al. [57], these categories are often interconnected, reflecting the holistic nature of nursing practice, where the assessment of competencies must be contextualized within clinical, relational, and organizational dimensions.

This variability reflects the influence of differing educational models and healthcare systems across countries. For instance, in Anglo-Saxon contexts, the emphasis tends to be on patient-centered approaches and clinical skills, whereas other regions may prioritize managerial or ethical–deontological competencies [58,59]. This differentiation aligns with the AACN position, which advocates for the definition of competencies that respond to local health needs while adhering to minimum global standards [15].

Despite these differences, a common thread among the frameworks is the attention to the multidimensional needs of individuals physical, psychological, social, and spiritual underscoring the centrality of person-centered care in nursing [21,32].

Notably, domains such as “safety” and “health promotion” are recurrent across frameworks, echoing the literature that identifies patient safety as one of the core competencies in healthcare [60].

To better highlight the commonalities and distinctions among the most frequently assessed areas, a brief synthesis was carried out regarding the predominance of domains and competencies. Across the analyzed frameworks, a consistent presence was noted for domains such as clinical reasoning, safety, communication, health promotion, and care coordination. These areas represent foundational expectations in nursing education worldwide and confirm a convergence toward shared professional priorities. While other competencies—such as leadership, digital literacy, and ethical decision-making—were present in some contexts, their distribution appeared more variable and context-dependent. This reflection, albeit qualitative, aligns with one of the core aims of this review: to identify prevailing trends and support a more coherent definition of common standards.

With respect to performance levels, although several theoretical models are identified including Bloom’s taxonomy, Miller’s pyramid, the national clinical judgment measurement model (NCJMM), and the Dublin descriptors there is substantial convergence in the expectations. All frameworks describe a developmental trajectory of competencies, from foundational knowledge to higher-order skills such as critical thinking and evaluation [54,55]. This progression reflects the recommendations of international nursing education guidelines, which emphasize the need to train nurses capable of exercising sound clinical judgment, particularly in complex and dynamic environments [4].

One of the most critical issues emerging from this review is the translation of these performance levels into actual assessment tools. Although theoretical frameworks are often well-structured, the assessment of competencies remains heavily weighted toward the cognitive domain. This finding, consistently reported across all included studies, is also corroborated by recent systematic reviews [61], which highlight ongoing challenges in evaluating psychomotor and relational skills.

The theoretical models referenced in the Methods section—namely the Job Demand–Resources Model, the Conservation of Resources Theory, and Trauma Theory—may also serve as useful interpretive tools to contextualize the institutional and emotional demands associated with licensure examinations. While not fully applied in this review, these models offer promising avenues to deepen the conceptual analysis of how candidates experience and respond to the pressures of certification systems across different settings. To enrich the interpretation of findings, future iterations of this review could benefit from anchoring the synthesis within a theoretical framework such as the Job Demand–Resources Model or the Conservation of Resources Theory. These models may offer additional explanatory value when examining how different licensure systems place varying cognitive, emotional, and institutional demands on new nursing graduates [23,24,25].

In order to analyze how different licensure systems influence the experience of newly graduated nurses, it is essential to consider not only the theoretical framework of the demands placed on them, but also the concrete tools through which these competencies are actually assessed. The multiple-choice question (MCQ) format is by far the most frequently used tool. While MCQs offer advantages in terms of standardization, efficiency, and cost-effectiveness [62], they are limited in their ability to assess complex reasoning and clinical judgment [63]. To address this gap, the Next-Generation NCLEX (NGN) has recently been introduced in the United States. Based on the NCJMM, the NGN incorporates complex item formats built around simulated clinical scenarios, aiming to capture both theoretical knowledge and decision-making processes [35,64]. This model represents a promising direction for other countries seeking to enhance the validity and reliability of licensure assessments.

In contrast, practical assessments such as bedside examinations, objective structured clinical examinations (OSCEs), or isolated skills tests remain underrepresented. This trend is consistent with the existing literature that identifies practical skill assessment as a resource-intensive process, requiring significant human, logistical, and ethical investment [65]. Nonetheless, several authors argue that practical skills assessment, particularly through simulation, is essential to prepare nurses for real-world clinical demands [66].

Interestingly, while the frameworks and competency domains exhibit high variability, the tools used for cognitive assessment show a greater degree of consistency. In centralized systems, such as that of United States, the use of standardized national exams contributes to this uniformity. In more decentralized contexts such as Europe, Africa and the Southeast Asian, where competencies are defined at the national or regional level, greater variability is observed [34,44,50].

To address this fragmentation, Europe has promoted initiatives aimed at harmonizing nursing education and assessment, such as the Tuning Project [17], and the EFN Competency Framework [19]. These initiatives offer a shared language for defining nursing competencies and may serve as a foundation for developing common evaluation criteria. Such standardization efforts are essential for fostering equity, transparency, and professional mobility within and across healthcare systems [18,38].

## 5. Strengths and Limitations

A key strength of this review lies in its systematic and comparative mapping of the international literature concerning nursing licensure examinations. By analyzing three fundamental dimensions competency frameworks and domains, expected performance levels, and assessment tools—the review offers a comprehensive and up-to-date perspective on the evaluation practices adopted in diverse geographical and cultural contexts. It also identified both convergences and divergences in assessment approaches, shedding light on the influence of regulatory, educational, and cultural factors in shaping national certification systems. These findings generate important insights for policy-makers, educators, and professional bodies, advocating for the design of shared, adaptable models to guide future licensure examinations.

Another notable strength is the applicative relevance of the results. The review supports the development of minimum common standards that could enhance the quality and equity of transitional pathways from education to professional practice. Furthermore, the inclusion of the literature from a range of international contexts contributes to the external validity of the findings.

However, this review also presents some limitations. First, the analysis was restricted to bachelor’s-level licensure examinations, excluding master’s level certifications and broader processes of competency assessment across the educational continuum. As such, the results apply only to first-level final examinations, omitting important evaluative dynamics present in advanced educational stages.

A further limitation to consider is the focus on the Bachelor of Science in Nursing (BSN) licensure examinations, which excludes other educational pathways such as the Associate Degree in Nursing (ADN) commonly found in the United States. Although this distinction was noted in the Limitations section regarding the exclusion of master’s level certifications, it is important to explicitly acknowledge that various educational levels may share the same licensure examination, potentially influencing the generalizability of the findings. Future research could expand the scope to include these diverse educational pathways and provide a more comprehensive understanding of the licensure process across all nursing qualification levels.

Second, the perspectives of students and evaluators were not included, nor were qualitative studies that could have enriched the findings with insights into subjective experiences, critical issues, and contextual nuances related to certification processes. This represents an important avenue for future research.

Lastly, despite a rigorous selection strategy, the available literature was heterogeneous in terms of terminology, framework structure, and completeness of reporting. Finally, despite a rigorous selection strategy, the available literature was heterogeneous in terms of terminology, framework structure, and documentation completeness. Furthermore, there was a notable predominance of studies from Anglo-Saxon countries, which may have influenced interpretation and limited the representation of other cultural and regional perspectives. Therefore, a potential limitation of this scoping review is the risk of publication bias, particularly due to the predominance of studies from the United States and the inclusion of literature primarily in English. This may have influenced the synthesis of findings and limited their generalizability to other regions and linguistic contexts.

## 6. Conclusions

This scoping review highlighted significant heterogeneity in the competency frameworks used internationally for nursing licensure examinations, both in terms of structural organization and the domains considered. Such variability reflects the diversity of healthcare systems, educational models, and socio-health priorities across countries.

Importantly, cultural and religious factors also play crucial roles in shaping nursing competencies and the content and delivery of licensure examinations. In some countries, cultural norms and religious laws influence healthcare practices, which consequently impact the definition of core competencies and assessment approaches. Recognizing these influences is essential to understanding international differences and fostering culturally sensitive competency frameworks.

Notwithstanding these differences, the review also identified key areas of convergence across the competency frameworks. Most models emphasize patient-centered care, clinical judgment, safety, and health promotion as essential domains for entry-level nursing practice. This suggests the existence of a shared set of core competencies that transcends national boundaries and may serve as a foundation for global standards. At the same time, differences in emphasis—such as the role of technology, communication, or ethical–legal aspects—reflect local priorities and contexts, highlighting the importance of maintaining both global alignment and local adaptability.

Nevertheless, a shared core emerges across frameworks, centered on the multidimensional needs of individuals and the promotion of health confirming the centrality of these concepts in defining global nursing competencies.

Despite differences in reference models for performance levels, most studies converge on a similar trajectory: beginning with foundational knowledge and advancing toward clinical judgment. This alignment underscores the growing emphasis on critical thinking and clinical reasoning as essential components of professional nursing practice.

From a methodological perspective, the most employed assessment tools are cognitive in nature, particularly multiple-choice questions (MCQs). In contrast, practical assessments remain underutilized, despite increasing evidence of their value for both educational and evaluative purposes. This imbalance underscores the urgent need to revise current evaluation models to better reflect the full spectrum of nursing competencies including relational, psychomotor, and ethical–deontological dimensions.

In light of these findings, there is a clear need to promote the harmonization of competency frameworks at the international level. Such efforts should aim to establish shared yet flexible standards that are responsive to local contexts while fostering comparability, equity, and professional mobility. In this regard, European initiatives such as the Tuning Nursing Project and the EFN Competency Framework represent promising models for advancing the quality and transparency of nursing education and certification processes.

Ultimately, ensuring equity and rigor in the evaluation of nursing competencies is a critical challenge for the future of the profession. Only through an integrated approach linking education, clinical practice, and professional regulation can we develop licensure systems that are relevant, robust, and aligned with the overarching goal of improving healthcare quality.

Future studies should aim to expand the scope of analysis by incorporating qualitative research, exploring advanced educational pathways, and evaluating alternative models of competency certification. Particular attention should be given to the development and implementation of integrated assessment systems that span the full trajectory of nursing education from academic training to professional practice.

## Figures and Tables

**Table 1:** Included studies (ordered alphabetically by author name).

Authors	Study Design	Population	Aim	Findings
Almarwani (2022) Saudi Arabia [29]	Quasi- experimental	109 undergraduate nursing students	To determine the effectiveness of implementing a Saudi Nursing Licensure Examination (SNLE) preparation course into nursing students’ curriculum	Implementing preparation programs, like SNLE, into the nursing curriculum seems feasible and effective for promoting nursing students’ readiness for the licensure examination and clinical practice
Amankwaa et al. (2015) Kumasi Africa occidental [30]	Descriptive cross- sectional	176 past nursing students	To identify whether education, sociodemographic characteristics, and nursing Cumulative Grade Point Average (CGPA) predict performance in the licensure examinations	Students’ previous education and demographic characteristics do not play a role in their performance in the licensure examinations
Athlin et al. (2012) Sweden [31]	Collaborative project	73 undergraduate nursing students	To describe the development and evaluation of the validity of a model for a national clinical final examination in bachelor’s nursing education	The model’s validity was confirmed, and since 2006, it has been adopted in the NCFE of 13 out of 25 Swedish universities
Baker (2019) Canada [32]	Pilot project	249 undergraduate nursing students	To develop and describe the Canadian Examination for Baccalaureate Nurses/examen canadien du baccalauréat en sciences infirmières (CEBN/ECBSI)	The psychometric statistical evaluation of the pilots tests indicated that the CEBN/ECBSI performed very well.
Benefiel (2011) United States (California) [33]	Non- experimental, quantitative, retrospective, correlational design	245 Bachelor students	(1) To analyze the relationship between preprogram, nursing program variables, and National Council Licensure Examination for Registered Nurses (NCLEX-RN) success and failure (2) To develop a model to predict success and failure on the NCLEX-RN	Assessment Technologies Institute (ATI) Test of Essential Academic Skills (TEAS) and gender, ethnicity, and age of students do not have a significant relationship to NCLEX-RN success
Efendi et al. (2018) Asia (Association of Southeast Asian Nations -ASEAN) [34]	Review	Not specified	(1) To compare information on nursing licensing examinations (NLE) across ASEAN countries (2) To describe the human resources required for a successful nursing workforce	NLE systems exist in all ASEAN Member States (AMSs) except Brunei, Vietnam, and Lao PDR. Nursing education systems, and language used in nursing examinations vary across countries. A qualified health workforce is above the threshold in some areas.
Forsman et al. (2019) Sweden [21]	Cross-sectional	179 undergraduate nursing students	(1) To identify clusters of graduating nursing students’ self-reported professional competence and their achievement on a national examination (2) To compare clusters of sample characteristics, perceptions of quality of the nursing program, and students’ general self-efficacy	Nursing students’ self-assessed competence differs from that assessed by examination.
Ignatavicius (2021) United States [35]	Descriptive	Not specified	To describe the National Council Licensure Exam (NCLEX), with a shift in focus from nursing process to clinical judgment	Clinical judgment definition and model, and new test item types were shared in case-based formats.
Lilja Andersson et al. (2013) Sweden [36]	Qualitative descriptive	577 undergraduate nursing students	To gain a deeper understanding of the students’ experiences of the strengths and weaknesses of the NCFE using open-ended questions	The NCFE is perceived by nursing students as a form of national quality assurance of nursing education, leading to improved learning and confirmation of skills and knowledge. To improve the NCFE, it is important to consider whether the questions in the written part should be raised and whether there is a need for greater standardization of bedside nursing
Msibi et al. (2020) Africa (Eswatini) [37]	Pilot	Not specified	To identify and to measure entry level competencies (knowledge, skills, attitudes, judgements) for nurses to practice safely and effectively in the Kingdom of Eswatini	Seven competency domains measured through multiple-choice questions: provision of quality care; information management systems emergency/trauma/disaster management; infection prevention and control; leadership and management; ethics/legal issues/professional conduct; prevention/treatment and care of HIV and AIDS, TB. Essential clinical skills are also assessed prior to obtaining licensure.
Pantaleo et al., (2023) Italy [38]	Multicentre observational prospective	Not specified	(1) To describe 47 Tuning competences evaluated during nursing licensure examination (2) To determine the performance levels of these competencies (3) To determine which clinical areas and settings these competences were assessing (4) To determine which types of tests were utilized for competence evaluation	In the nursing licensure exam held from 2017 to 2019 in 4 universities, Tuning competencies were requested 7522 times. The prevalent competencies were those associated with domain 2: ‘Nursing practice and clinical decision making’. The level of performance most required in cognitive tests was the autonomy of judgment, and tests concerned non-communicable diseases and, hospitalized adult patients
Petrovic et al. (2019) Canada [39]	Descriptive- Sociological	Not specified	To describe the institutional processes in adopting the NCLEX-RN in Canada	This manuscript questions the appropriateness of NCLEX-RN in the Canadian context. The strategies discussed may have utility in other contexts and countries facing similar changes.
Pike et al. (2019) Canada [40]	Cross- correlational	234 undergraduate nursing students	To describe nursing graduates’ performance on the first-time writing of the NCLEX-RN in NL and their relationships with candidate demographic and academic variables.	The strongest predictor of NCLEX-RN was the number of questions answered on the exam. The lower the number of questions answered, the higher the probability of passing the NCLEX-RN.
Pressler & Kenner (2012) United States [41]	Descriptive	Not specified	Identify instructional strategies to support student success on the NCLEX-RN exam	To maintain the level of competency desired, students must undergo ongoing evaluation. Deans should make a concerted effort to ensure that ongoing assessments of students’ success in such standardized review examinations are completed.
Reid (2000) America Commonwealth Caribbean [42]	Descriptive	Not specified	Analyze the regional nursing licensure examination model in the Caribbean Commonwealth countries. The author describes the exam’s characteristics, history, and implementation, drawing on administrative data and official documentation.	The model is accepted and is based on mutually agreed competencies for the registered nurse to practice in the region.
Singh (2017) United States [43]	Exploratory and comparative	148 Undergraduate nursing students	To explore a computerized adaptive testing program and Pass Point and to identify any predictors for NCLEX-RN success on first attempt	The number of quizzes completed in the Pass Point was the only statistically significant variable and positively correlated with the likelihood of being unsuccessful
Strube- Lahmann et al. (2016) Germany [44]	Descriptive comparative	43,242 nursing student	To analyze the results of final examinations for nursing education in Berlin between 2008 and 2013. The study evaluates the differences in exam scores between centrally (Zentral) and non-centrally (Dezentral) organized examinations, considering the different models of professional nursing education.	In nursing schools with a traditional approach to education, there was a big difference in grades between written and oral/practical exams. Standardization of oral and practical exams should be initiated to ensure greater comparability between different educational institutions
Tahir et al. (2021) Indonesian [45]	Cross- correlational	727 bachelor nurses	To explore the predictor factors associated with the nurses passing the INCE	Completing a standard internship program was the main predictor for passing the INCE. Nursing universities need to provide internship programs based on the national standard. Additional research is needed on other associated factors
Wendt & Brown (2000) United States [46]	Expert opinions	Not specified	To investigate the development of different item types and use of different testing methodologies in NCLEX(R) examination, based on the practice analysis and expert opinion results	The National Council’s Examination Committee recommended that no change be made to the test plan structure or the percentage of questions allocated to each Client Needs subcategory
Wendt (2001) United States [47]	Descriptive comparative	Not specified	To map the geriatric competencies identified by the American Association of Colleges of Nursing (AACN) and the John A. Hartford Foundation Institute for Geriatric Nursing to the test plan of NCLEX RN	Geriatric nursing skills cannot be directly mapped to a category or subcategory of customer needs of the NCLEX-RN test plan. No one-to-one correspondence exists between a geriatric nursing skill and a test plan category or subcategory
Wendt (2003) United States [48].	Descriptive	Not specified	To describe the changes in the 2004 NCLEX-RN® Test Plan and to provide information about the alternate item formats that are being developed for the NCLEX examination	The format of alternative items in NCLEX RN is a potential vehicle for determining cognitive processing development
Wendt & Kenny (2007) United States [49]	Descriptive	Not specified	To illustrate the strategies adopted by the National Council of State Boards of Nursing (NCSBN) to update and maintain the NCLEX-RN exam.	Regular monitoring allows the NCLEX-RN to be dynamically adapted, improving the quality and validity of the exam as a measure of professional preparation.
Yim & Shin (2020) Korea [50].	Descriptive- explorative	Panel of 16 nursing professors	To establish criteria for mock KNLE exams using the Angoff method and to analyze the results	A performance level description (PLD) was established to reflect the minimum competency level of new nurses

Legend: AACN = American Association of Colleges of Nursing; AIDS = Acquired Immune Deficiency Syndrome; AMSs = ASEAN Member States; ASEAN = Association of Southeast Asian Nations; ATI = Assessment Technologies Institute; CEBN/ECBSI = Canadian Examination for Baccalaureate Nurses/l’Examen Canadien du Baccalaureate en Sciences Infirmières; NCLEX-RN = National Council Licensure Examination for Registered Nurses; CGPA = Cumulative Grade Point Average; ENC = Eswatini Nursing Council; GSE = general self-efficacy: HIV = Human Immunodeficiency Virus, INCE = Indonesian Nursing Competency Examination; KNLE = Korean Nursing Licensing Examination; NCFE = Swedish National Clinical Final Examination; NCLEX = National Council Licensure Examination; NCLEX-RNA = National Council Licensure Examination; NCSBN = National Council of State Boards of Nursing; NGN = Next-Generation NCLEX; NL = Newfoundland and Labrador; NLE = Nursing Licensing Exam; PLD = performance level description. RN = Registered Nurse; SNLE = Saudi Nursing Licensure Examination; TB = tuberculosis; TEAS = Test of Essential Academic Skills; USA = United States of America.

**Table 2:** Framework and domains of competencies.

Framework Competencies	Study	Denomination of Nursing Licensure	Domains of Competencies	Competence
Client Needs in all healthcare settings. (a)	Benefiel (2011) [33] Ignatavicius (2021) United States (California) [35]	NCLEX-RN NCLEX (NGN)	**4 Categories of nursing** 1. Safe and effective care environment 2. Physiological integrity 3. Psychosocial integrity 4. Health promotion	**Management of Care** ● Coordinate and plan nursing care effectively. ● Manage healthcare resources and personnel. ● Advocate for patients and protect their rights. ● Educate and support patients and families. ● Accurate documentation and management of clinical information. ● Interprofessional collaboration. ● Evaluate and improve quality of care. **Safety and Infection Control** ● Apply infection prevention protocols (e.g., hand hygiene, PPE use). ● Identify and manage patient safety risks. ● Monitor and prevent accidents and injuries. ● Safely manage medical equipment. ● Educate patients and staff on safety practices. ● Isolate and control transmissible infections. **Health Promotion & Maintenance** ● Assess health status and risk factors. ● Provide health education and counseling for disease prevention. ● Promote healthy lifestyles (nutrition, exercise, smoking cessation). ● Support adherence to screening programs and vaccinations. ● Monitor growth and development (pediatrics, geriatrics). ● Support chronic disease management. **Physiological Integrity** ● Conduct comprehensive assessment of patient’s physiological status (vital signs, diagnostic tests). ● Manage acute and chronic conditions compromising physiological integrity. ● Monitor and intervene in electrolyte, nutritional, and fluid balance disorders. ● Manage respiratory and cardiovascular function (e.g., ventilatory support, ECG monitoring). ● Assess and manage pain and inflammatory responses. ● Support neurological and sensory functions (e.g., prevent neurological complications). ● Implement interventions to maintain or restore physiological integrity. **Psychosocial Integrity** ● Assess psychological and social status of the patient. ● Provide emotional support and counseling. ● Identify and manage psychiatric and behavioral problems. ● Promote psychological adaptation to illness. ● Support management of stress, anxiety, and depression. ● Collaborate with mental health professionals. **Basic Care & Comfort** ● Assist with activities of daily living (hygiene, nutrition, mobility). ● Monitor and maintain patient comfort. ● Manage pain and suffering. ● Support communication and social interaction. ● Prevent pressure ulcers. ● Assist in managing basic physiological problems (e.g., elimination, breathing). **Pharmacological & Parenteral Therapies** ● Safely administer oral, parenteral, and topical medications. ● Evaluate therapeutic and adverse effects of medications. ● Monitor drug interactions. ● Manage intravenous therapy and other parenteral treatments. ● Educate patients on medication therapy. ● Understand indications, contraindications, and dosages. **Reduction in Risk Potential** ● Early identification of complications and risk factors. ● Monitor vital signs and clinical parameters. ● Prevent falls, injuries, and clinical complications. ● Apply interventions to reduce risks (e.g., thromboembolism prophylaxis). ● Educate patients and families on risk factors. ● Manage emergencies and respond promptly to changes in clinical status. **Physiological Adaptation** ● Assess and monitor altered physiological conditions. ● Manage patients with acute and chronic illnesses. ● Support compromised vital functions (respiratory, cardiac, renal, etc.). ● Interpret laboratory and diagnostic test results. ● Implement interventions to promote adaptation and stabilization. ● Collaborate in planning rehabilitative treatments.
	Pike et al. (2019) Canada (Newfoundland and Labrador) [40]	NCLEX-RN	**8.** **Domains** 1. Management care 2. Safety and infection control 3. Health promotion and maintenance 4. Psychosocial integrity 5. Basic care and comfort 6. Pharmacological and parenteral therapies 7. Reduction in risk potential 8. Physiological adaptation	
	Pressler & Kenner (2012) United States [41] Singh (2017) United States [43] Wendt (2003) [48] Wendt & Kenny (2007) United States [49]		**4 Domains and 6 sub-categories** 1. Safe And Effective Care Environment: - management of care - safety and infection control 2. Health Promotion And Maintenance 3. Psychosocial Integrity 4. Physiological Integrity: - basic care and comfort - pharmacological and parenteral therapies - reduction in risk potential, - physiological adaptation.	
	Wendt & Brown (2000) United States [46]	NCLEX(R)	**5 domains and 10 subcategories (2 for each category)** 1. Safe, Effective Care Environment: - Management of care - Safety and infection control. 2. Health Promotion And Maintenance: - growth and development thorough the life span - prevention and early detection of disease 3. Psychological Integrity: - coping and adaptation - Psychological adaptation 4. Physiological Integrity: - basic care and comfort - pharmacological and parenteral 5. Therapies: - reduction in risk potential - physiological adaptation	
	Wendt (2001) United States [47]		**5 domains and 10 subcategories (2 for each category)** 1. Safe, Effective Care Environment: - Management of care - Safety and infection control. 2. Health Promotion And Maintenance: - growth and development thorough the life span - prevention and early detection of disease 3. Psychological Integrity: - coping and adaptation - Psychological adaptation 4. Physiological Integrity: - basic care and comfort - pharmacological and parenteral 5. Therapies: - reduction in risk potential - physiological adaptation	
2. Saudi Nursing Licensing Exam Guideline (a)	Almarwani (2022) Saudi Arabia [29]	SNLE	**5 domains** 1. Adult Nursing care 2. Maternity and child care 3. Psychiatric Nursing care 4. Nursing research 5. Complete instrument	**Adult Nursing Care** ● comprehensive assessment and management of adult patients’ health conditions. ● Implementation of care plans for acute and chronic diseases. ● Monitoring vital signs, administering medications, and managing therapies. ● Patient education on disease prevention, management, and health promotion. ● Support in rehabilitation and recovery processes. ● Management of complications and emergencies in adult patients. ● Coordination of multidisciplinary care. **Maternity and Child Care** ● Care of women during pregnancy, labor, delivery, and postpartum period. ● Monitoring fetal and maternal well-being. ● Providing neonatal care and supporting breastfeeding. ● Health education for mothers and families on prenatal and postnatal care. ● Management of common pediatric illnesses and developmental monitoring. ● Supporting growth, nutrition, and immunization schedules in children. ● Identifying and managing high-risk pregnancies and pediatric conditions. **Psychiatric Nursing Care** ● Assessment of mental health status and psychiatric conditions. ● Implementation of therapeutic communication and counseling techniques. ● Management of patients with mental illness, including medication administration and monitoring. ● Crisis intervention and management of behavioral emergencies. ● Promotion of psychosocial rehabilitation and support for patient autonomy. ● Collaboration with multidisciplinary mental health teams. ● Education of patients and families on mental health conditions and coping strategies **Nursing Research** ● Understanding of basic research principles and methodologies ● Ability to critically appraise nursing research literature. ● Participation in research activities to improve nursing practice support for patient autonomy.
				● Collaboration with multidisciplinary mental health teams. ● Education of patients and families on mental health conditions and coping strategies Nursing Research ● Ability to critically appraise nursing research literature. ● Participation in research activities to improve nursing practice. ● practice. ● Application of evidence-based practice in clinical settings. ● Data collection, analysis, and interpretation relevant to nursing care. ● Dissemination of research findings to healthcare teams. ● Ethical considerations in nursing **Complete Instrument** ● Comprehensive application of nursing knowledge and skills across all domains. ● Integration of theory and practice in clinical decision-making. ● Demonstration of professional attitudes and ethical nursing practice. ● Time management and prioritization of patient care tasks. ● Effective communication with patients, families, and healthcare team. ● Adherence to safety standards and infection control measures. ● Use of clinical judgment to deliver holistic and patient-centered care
3. Standards of the Nursing and Midwifery Council (a)	Amankwaa et al. (2015) Kumasi Africa Occidental [30]	NMC-LE	**6 Domains for the theoretical component:** 1. Medical Nursing 2. Surgical Nursing 3. Mental Health Nursing 4. Pediatric Nursing 5. Public Health Nursing 6. Obstetric Nursing	**Medical Nursing** ● Assessment and management of patients with medical conditions. ● Implementation of nursing interventions to support recovery and manage symptoms. ● Monitoring patient responses and adjusting care plans accordingly. ● Administration and management of medications and treatments. ● Patient education on disease processes and self-care. ● Coordination of multidisciplinary care for complex medical needs. **Surgical Nursing** ● Preoperative, intraoperative, and postoperative nursing care. ● Management of surgical wounds and prevention of infections. ● Pain management and monitoring for complications. ● Patient education on surgical procedures and recovery. ● Support for mobility and rehabilitation post-surgery. ● Coordination with surgical teams and effective communication. **Mental Health Nursing** ● Assessment of mental health status and psychosocial needs. ● Development and implementation of therapeutic care plans. ● Crisis intervention and management of behavioral emergencies. ● Medication administration and monitoring of psychiatric treatments. ● Counseling and support for patients and families. ● Collaboration with mental health professionals and community resources. **Pediatric Nursing** ● Comprehensive care of infants, children, and adolescents. ● Monitoring growth and development milestones. ● Administration of pediatric medications and immunizations. ● Support for families and education on child health and safety. ● Management of common pediatric illnesses and chronic conditions. ● Coordination of care with pediatric specialists. **Public Health Nursing** ● Health promotion and disease prevention at the community level. ● Conducting health assessments and screenings. ● Implementation of vaccination and immunization programs. ● Education on healthy lifestyles and risk reduction. ● Collaboration with public health agencies and community organizations. ● Advocacy for vulnerable populations and health equity. **Obstetric Nursing** ● Care of women during pregnancy, labor, delivery, and postpartum. ● Monitoring maternal and fetal health. ● Support for normal and high-risk pregnancies. ● Education on childbirth, breastfeeding, and newborn care. ● Management of complications in pregnancy and childbirth. ● Collaboration with multidisciplinary maternity care teams.
4. The National Board of Health and Welfare Competence Declaration for Registered Nurses In Swedish (a)	Athlin et al. (2012) Sweden [31]	NCFE	**4 Domains** Theoretical and practical nursing Research Development and teaching Leadership	**Theoretical and Practical Nursing** ● Comprehensive application of nursing theories and evidence-based practices in clinical care. ● Assessment, planning, implementation, and evaluation of nursing care tailored to patient needs. ● Mastery of practical nursing skills across diverse healthcare settings. ● Integration of holistic care approaches, including physical, psychological, social, and cultural aspects. ● Safe administration of medications and therapies. ● Critical thinking and clinical decision-making to ensure patient safety and quality care **Research** ● Understanding and application of nursing research principles and methodologies. ● Ability to critically appraise and utilize research findings to improve nursing practice. ● Participation in research projects and quality improvement initiatives. ● Promotion and implementation of evidence-based practice in healthcare settings. ● Ethical considerations and adherence to regulations in nursing research. **Development and Teaching** ● Development and implementation of nursing education and training programs. ● Facilitation of learning for patients, families, colleagues, and students. ● Promotion of professional development and lifelong learning within nursing teams. ● Contribution to the development of nursing practice guidelines and protocols. ● Ability to communicate complex nursing concepts clearly and effectively. **Leadership** ● Leadership and management of nursing teams and healthcare resources ● Coordination and delegation of nursing tasks in clinical settings ● Advocacy for patients and professional nursing interests. ● Facilitation of interprofessional collaboration and communication. ● Strategic planning and decision-making to enhance nursing practice and patient outcome
5. Tuning nursing specific competences. (a)	Pantaleo et al. (2023) Italy [38]	NLE	**5 Domains** 1. Competences associated with professional values and the role of the nurse (6 items); 2. competences associated with nursing practice and clinical decision-making (14 items); 3. knowledge and cognitive competences (10 items); 4. communication and interpersonal competences including communication technologies (9 items); 5. leadership, management, and group dynamics management competences (8 items).	See file Tuning Project: Nursing Specific Competences aviable from http://www.unideusto.org/tuningeu/competences/specific/nursing.html (accessed on 08 October 2024)
6. Canadian Association of Schools of Nursing National Framework for Baccalaureate Education (a)	Baker (2019) Canada [32]	CEBN/ECBSI	**6 domains** 1. Knowledge 2. Research 3. Entry-level practice (6 areas: new-born; infant child, and adolescent; adult; childbearing person; older person; and end-of-life) 4. Communication and collaboration 5. Leadership 6. Professionalism	**Knowledge** ● Integration of foundational knowledge from nursing, health sciences, social sciences, and humanities. ● Application of theoretical and scientific principles to nursing practice. ● Use of critical thinking and clinical reasoning to inform decision-making. ● Understanding of ethical, legal, and regulatory frameworks in healthcare. ● Lifelong learning and engagement with emerging knowledge and innovations. **Research** ● Understanding of research principles, methods, and evidence appraisal. ● Participation in the generation and application of nursing research. ● Integration of research findings into evidence-informed practice. ● Contribution to quality improvement and practice development. ● Ethical use and dissemination of research in clinical settings. **Entry-Level Practice** a. Newborn ● Conduct comprehensive newborn assessments. ● Support family-centered care and breastfeeding. ● Monitor neonatal adaptation and risk factors. b. Infant, Child, and Adolescent ● Promote healthy growth and development. ● Deliver age-appropriate care and education. ● Identify and respond to common pediatric conditions. c. Adult ● Assess and manage acute and chronic health conditions. ● Deliver individualized and evidence-based care. ● Promote self-management and rehabilitation. d. Childbearing Person ● Support care during preconception, pregnancy, labor, and postpartum. ● Monitor maternal and fetal health. ● Provide education on childbirth and newborn care. e. Older Person ● Promote healthy aging and autonomy. ● Recognize and manage geriatric syndromes. ● Support caregivers and end-of-life transitions f. End-of-Life ● Provide palliative and hospice care. ● Manage pain and symptoms compassionately. ● Offer emotional and spiritual support to patients and families. **Communication and Collaboration** ● Use of effective verbal, non-verbal, and written communication. ● Therapeutic communication with individuals, families, and communities. ● Collaboration within interprofessional healthcare teams. ● Conflict resolution and negotiation in clinical settings. ● Cultural safety and sensitivity in all interactions **Leadership** ● Demonstration of accountability and initiative in clinical practice. ● Advocacy for patients, families, and health system improvement. ● Participation in policy development and system change. ● Application of leadership principles to guide nursing teams. ● Engagement in mentorship and role modeling. **Professionalism** ● Adherence to professional standards, codes of ethics, and legal responsibilities. ● Commitment to patient-centered care, respect, and equity. ● Maintenance of competence and reflective practice. ● Accountability in decision-making and actions. ● Promotion of the nursing profession and public trust.
7. ASEAN Joint Coordinating Committee on Nursing (a)	Efendi et al. (2018) Asia (Association of Southeast Asian Nations -ASEAN) [34]	NLE	**5 domains** 1. Ethics and legal practices 2. Education and research 3. Leadership and management 4. Professional nursing practices 5. Professional, personal and quality development	**Ethics and Legal Practices** ● Demonstrate ethical reasoning and adherence to professional codes of conduct. ● Uphold human rights, dignity, and patient autonomy. ● Apply national and international laws and regulations in nursing practice. ● Ensure confidentiality, informed consent, and privacy. ● Manage ethical dilemmas and report unsafe or unethical practices. **Education and Research** ● continuous education and professional development. ● Promote evidence-based practice through the integration of research findings. ● Support and engage in nursing research activities. ● Educate patients, families, and communities on health-related issues. ● Contribute to the development of nursing curricula and educational programs. **Leadership and Management** ● Lead and supervise nursing teams to ensure high-quality care. ● Apply principles of management, delegation, and coordination. ● Engage in decision-making and strategic planning. ● Promote a safe and ethical work environment. ● Contribute to policy development and system improvement. **Professional Nursing Practices** ● Deliver comprehensive, culturally sensitive, and patient-centered care. ● Perform nursing assessments, develop care plans, and evaluate outcomes. ● Provide care across the lifespan and in a variety of healthcare settings. ● Ensure safety, infection control, and risk management. ● Collaborate with interdisciplinary teams to meet health needs. **Professional, Personal, and Quality Development** ● Maintain and improve personal and professional competencies. ● Reflect on practice and seek opportunities for growth. ● Demonstrate accountability, responsibility, and commitment to the profession. ● Engage in quality assurance and improvement initiatives. ● Promote lifelong learning and self-awareness in nursing roles
8. Quality and Safety Education for Nurses (a)	Forsman et al. (2019) Sweden [21]	NCFE	**6 domains** 1. Patient-centered care 2. Teamwork and collaboration 3. Evidence-based practice 4. Quality improvement 5. Safety 6. Informatics	**Patient-Centered Care** ● Recognize the patient or designee as the source of control and full partner in providing compassionate and coordinated care. ● Respect patient preferences, values, and needs. ● Involve patients and families in decision-making processes. **Teamwork and Collaboration** ● Function effectively within nursing and interprofessional teams. ● Foster open communication, mutual respect, and shared decision-making. ● Use strategies to manage conflict and enhance team performance. **Evidence-Based Practice** ● Integrate best current evidence with clinical expertise and patient/family preferences for optimal care. ● Critically appraise and apply scientific evidence. ● Promote the use of evidence in everyday clinical decision-making. **Quality Improvement** ● Use data to monitor outcomes and improve nursing care processes. ● Participate in quality improvement initiatives. ● Identify gaps in care and suggest practical solutions for enhancement. **Safety** ● Minimize risk of harm to patients and providers through system effectiveness and individual performance. ● Recognize and report errors or near misses. ● Use safety tools (e.g., checklists, protocols) and promote a safety culture. **Informatics** ● Use information and technology to communicate, manage knowledge, mitigate errors, and support decision-making. ● Understand electronic health records (EHRs) and clinical decision support systems. ● Maintain confidentiality and data security.
9. ENC Competency Framework Client-Centered (a)	Msibi et al. (2020) Africa (Eswatini) [37]	ETP examination	**7.** **domains** 1 The provision of quality care 2 Information management systems 3 Emergency, trauma, and disaster management 4 Infection prevention and control 5 Leadership and management 6 Ethics, legal issues, and professional conduct 7 Prevention, treatment, and care—HIV/AIDS and TB	**The Provision of Quality Care** ● Deliver safe, effective, and compassionate care centered on the needs, preferences, and values of clients. ● Conduct comprehensive assessments and implement individualized care plans. ● Continuously evaluate care outcomes to ensure high standards. ● Promote continuity of care across settings and care levels. ● Apply evidence-based interventions in clinical practice **Information Management Systems** ● Utilize health information systems to document, retrieve, and analyze patient data. ● Ensure accuracy, confidentiality, and security in handling digital health records. ● Use clinical decision support tools to enhance care quality. ● Apply informatics to support evidence-based nursing practice **Emergency, Trauma, and Disaster Management** ● espond effectively to medical emergencies, trauma cases, and disasters. ● Triage and prioritize care under pressure and in resource-limited environments. ● Collaborate with multidisciplinary teams during crises. ● Apply principles of emergency preparedness and recovery. ● Engage in simulation and training for emergency response. **Infection Prevention and Control** ● Implement standard precautions and transmission-based precautions. ● Monitor and reduce healthcare-associated infections (HAIs). ● Educate patients and staff on IPC best practices. ● Participate in surveillance, reporting, and outbreak response outbreak response outbreak response. ● Promote hand hygiene, aseptic techniques, and environmental sanitation. **Leadership and Management** ● Lead nursing teams and manage resources to optimize care delivery. ● Supervise, mentor, and support staff development. ● Apply management principles in planning, organizing, and evaluating care. ● Promote teamwork, communication, and accountability. ● Advocate for policy and system improvements in healthcare. **Ethics, Legal Issues, and Professional Conduct** ● Practice in accordance with professional codes of ethics and legal standards. ● Demonstrate integrity, accountability, and respect in all professional interactions. ● Recognize and address ethical dilemmas and legal concerns. ● Promote informed consent, autonomy, and patient rights. ● Report misconduct and contribute to ethical workplace culture. **Prevention, Treatment, and Care—HIV/AIDS and TB** ❖ Provide stigma-free, evidence-based care for individuals with HIV/AIDS and TB. ❖ Conduct screening, counseling, and health education. ❖ Monitor adherence to antiretroviral therapy and TB treatment regimens. ❖ Prevent transmission through infection control and community outreach. ❖ Support patients and families through psychosocial and nutritional care
10. Entry to Practice Competencies Client-Centered (a)	Petrovic et al. (2019) Canada [39]	CRNE/NCLEX-RN	**6 domains** 1. Professional responsibility and accountability 2. (23 competencies) 3. Knowledge-based practice: specialized body of Knowledge (12 competencies) 4. Knowledge-based practice: competent application of knowledge (39 competencies) 5. Ethical Practice (12 competencies) 6. Service to the public (8 competencies) 7. Self-regulation (6 competencies)	**Not specified**
11. Bluprint and curricula of all participating Schools of nursing	Reid (2000) America Commonwealth Caribbean [42]	Regional Examination for Nurse Registration	The health needs of each country and the General Nursing Councils’ definition of professional nursing practice.	**Not specified**
12. Minimum practice standards of Association of Indonesian Nurse Education Centre (a)	Tahir et al. (2021) Indonesian [45]	INCE	Not specified	**Not specified**
13. Angoff Method (b)	Yim & Shin (2020) Korea [50]	KNLE	**8** **domains** 1 Adult Nursing 2 Fundamental Nursing 3 Maternal Nursing 4 Pediatric Nursing 5 Community Nursing 6 Psychiatric Nursing 7 Nursing Management 8 Medical Health Legislation	**Adult Nursing** ● Assessment, planning, and delivery of care to adult patients. ● Management of chronic and acute illnesses. ● Application of critical thinking and evidence-based practice in adult care settings **Fundamental Nursing** ● Assessment, planning, and delivery of care to adult patients. ● Management of chronic and acute illnesses. ● Application of critical thinking and evidence-based practice in adult care settings. **Maternal Nursing** ● Care of women during pregnancy, labor, and postpartum. ● Fetal monitoring, prenatal education, and obstetric emergencies. ● Newborn assessment and maternal well-being. **Pediatric Nursing** ● Growth and development stages from infancy to adolescence. ● Communication with children and families. ● Pediatric disease management and immunization protocols. **Community Nursing** ● Health promotion and disease prevention at the population level. ● Home healthcare, school nursing, and public health interventions. ● Epidemiology and surveillance within community settings **Psychiatric Nursing** ● Mental health assessment and intervention. ● Therapeutic communication and management of psychiatric disorders. ● Crisis intervention and promotion of psychosocial integrity **Nursing Management** ● Leadership, delegation, and supervision in clinical settings. ● Resource and personnel management. ● Quality improvement and policy implementation **Medical Health Legislation** ● Knowledge of health-related laws and nursing regulations. ● Patient rights, informed consent, confidentiality ● Ethical and legal responsibilities in practice.
			
Not specified	Lilja Andersson et al. (2013) Sweden [36]	NCFE	Not specified	Not specified
	Strube-Lahmann et al. (2016) Germany [44]	NLE	Health and Nursing care Health and PaediatricNursing, Geriatric care	Not specified

Legend: (a) = officially adopted national or institutional competency models; (b) = frameworks used by researchers for analytical or comparative purposes; not specified = nature of the framework’s adoption was not explicitly stated in the original source; AIDS = Acquired Immune Deficiency Syndrome; AINEC = Association of Indonesian Nurse Education Centre; AJCCN = ASEAN Joint Coordinating Committee on Nursing; ASEAN = Association of Southeast Asian Nations; CASN = Canadian Association of Schools of Nursing; CEBN/ECBSI = Canadian Examination for Baccalaureate Nurses/l’Examen Canadien du Baccalaureate en Sciences Infirmières; CRNE = Canadian Registered Nursing Exam; ENC = Eswatini Nursing Council; ETP = Entry to Practice Competencies; ETPC = Entry to Practice Competencies Client-Centered; HIV = Human Immunodeficiency Virus; INCE = Indonesian Nursing Competency Examination; KNLE = Korean Nursing Licensing Examination; NCFE = Swedish National Clinical Final Examination; NCLEX = National Council Licensure Examination; NCLEX-RN = National Council Licensure Examination for Registered Nurses; NGN = Next-Generation; NLE = Nursing Licensing Exam; NMC = Nursing and Midwifery Council; NMC-LE = Nursing and Midwifery Council- Licensure Examination; QSEN = Quality and Safety Education for Nurses; SNLE = Saudi Nursing Licensure Examination; TB = tuberculosis.

**Table 3:** Expected performance levels.

Denomination of Descriptors of Performance Levels	Expected Performance Levels
Bloom’s taxonomy	6. Levels *1*. *Remember* - ability to recall information from memory *2*. *Understand* - ability to demonstrate comprehension of information *3*. *Apply* - ability to use information in new situations *4*. *Analyze* - ability to break down information into smaller parts to understand the relationships between them *5*. *Evaluate* - ability to requires making judgments based on criteria and standards *6*. *Create* - ability to put together information to form something new [33] 6 Levels *1. Knowledge* - ability to recall and repeat specific information, such as definitions, facts, and concepts. *2. Comprehension* - ability to understand the provided information, interpret it, and explain it. *3. Application* - ability to apply acquired knowledge to solve problems or situations in different contexts. *4. Analysis* - ability to interpret and analyze acquired information. *5. Synthesis* - ability to create something new by combining information in an original and creative way. *6. Evaluation* - ability to evaluate information or situations based on specific criteria [46,47,48,49].
2. Miller’s Pyramide	4 Levels *1. Learner’s Knowing* knowing the principles/content of basic scientific knowledge. *2. Knowing How* ability to use knowledge in the acquisition, analysis, and interpretation of data. *3. Showing How* ability to demonstrate integration of knowledge and skills. *4. Doing* ability to evaluate clinical performance in real practical contexts [21,31,32]
3. National Clinical Judgment Measurement Model of Tanner (NCJMM)	4 Levels *1. Noticing The Client’s Condition And Clinical Situation* - Recognize cues, ability to detect signs and symptoms related to a specific clinical condition of the patient. *2. Interpreting* - Analyze cues, ability to analyze signs and symptoms to justify the choice of the nursing diagnosis. *3. Responding* - Prioritize hypotheses, ability to prioritize the client’s potential needs. - Generate solutions, ability to determine priority hypotheses to plan and anticipate specific actions to achieve desirable outcomes. - Take action, ability to implement relevant interventions with the objective of achieving the goal. *4. Reflecting* - evaluate outcomes, ability to evaluate the actual outcomes achieved by the client and compare them with the expected outcomes [35]
4. Dublin Descriptors	5 descriptors *1*. *Knowledge And Understanding* - Ability to know and memorize concepts. *2*. *Applying Knowledge And Understanding* - Ability to interpret data, signs, symptoms, and patient needs in a specific clinical situation. *3*. *Making Judgements* - Ability to solve simple or complex problems, plan, and define appropriate care interventions. *4*. *Communication Skills* - Ability to engage in two-way communication and establish a therapeutic relationship as an empathetic interaction between nurses and patients. *5*. *Learning Skills* - Ability to develop the capacity to question the practice of one’s own activity [38]
5. Standard Clinical Competencies Record Book (*SCCRB)*	*Knowledge* - Unspecified ability [37]
6. Not specified framework but the performance levels are only listed	*Knowledge* [34,40,43,45]. *Knowledge And Understanding* [41] 4 Levels *1*. *Knowledge* *2*. *Transfer* *3*. *Reflection* *4*. *Application* [44] 4 Levels: *1. Knowledge Abilities In Interpretation* *2. Analysis* *3. Decision-Making* *4. Reasoning And Problem Solving Skills* [29]. 3 Levels 1. *The Assessment Of The Patient’s Needs And Problems, Analyses And Planning* *2. Implementation And Evaluation Of Nursing Activities* *3. Reflections And Final Judgment* [36]
7. Not specified framework and performance levels	[30,39,42,50]

Legend: NCJMM = National Clinical Judgment Measurement Model; SCCRB = Standard Clinical Competencies Record Book.

**Table 4:** Types of tools used for competencies assessment.

Theoretical or Cognitive Test	
Types of tools	Description of theoretical test (specific for each study of the scoping review)
1. Multiple-choice question (*MCQ*)	150 multiple-choice questions for each question, nursing students must select the best response out of 4 possible answer [29] ▪ Unknown number of tests for 6 nursing area [30] ▪ Unknown number of test with closed questions [38,39] ▪ 200 multiple-choice questions on each of three ex amination forms. The questions test: knowledge (10%), application (40–50%), clinical reasoning and clinical judgment (40–50%) [32] ▪ 75 to 295 tests (CAT) [33,40,41,43,46,47,48,49,50] ▪ Unknown number of tests for Indonesian, Thailand, Philippines, Singapore, Malaysia and Cambodia [34] ▪ The test elements are based on the cases in the NGN. Stand-alone scenarios, ongoing case studies include 3 elements: a clinical scenario six test items corresponding to the six cognitive skills of the NCJMM Various new types of multiple-choice test items. The new evidence for the conduct cases can be classified into 5 categories with multiple subtypes: 1. multiple response and 2. multiple-choice, candidates choose the correct answer out of 4 possible options 3. highlight, candidates write down the relevant patient data in a chart 4. drop-down, presents 3 types of data entry: - drag and drop rationale, the student can insert a sentence into the text by choosing it from a token. - drop-down table, the student chooses a term or phrase from a drop-down menu - drop-down cloze, the student completes an entire paragraph by choosing one or more sentences from the drop-down menu 5. drag and drop, the student can click on a virtual object (such as a window or an icon) to drag it to another position (drag), where it is released (drop). This last option has 2 subtypes of questions: drag-and-drop cloze questions and drag-and-drop rationale questions [35] ▪ Unknown number of multiple-choice questions to perform essential procedures and record in the SCCRB. It is co-signed by clinical preceptors after the student has demonstrated competency in that procedure [37] ▪ Multi-choice items based on case study scenario [46]
2. Modified essay questions (*MEQs)*	▪ To pass, students need 33 points of a possible 50 and two questions about drug calculation must be correctly answered [21]
3. Multiple-choice question and modified essay questions	▪ 4 papers, 158 items: ● Paper I: clinical nursing, essay-type test items—4 items, ● Paper II: clinical nursing, objective-type test items—100 items, ● Paper III: functional nursing, essay-type test items—4 items, ● Paper IV: functional nursing, objective-type items—50 items [42]
4. Test with open question	Unknown number of tests with open questions [38]
5. Resolution of real or simulated cases	▪ 4 h test on the resolution of two real cases [31] ▪ The written test is problem-solving in character and consists of two patient cases describing realistic care situations [36] ▪ Oral and/or written discussion of clinical cases [38] ▪ Computer based test, 180 questions within 180 min based on real patient cases (the patient’s illness, medical history, etc.) and the care that is provided until the patient’s condition is resolved [45] ▪ Oral exams are developed by each individual nursing school or university and conducted without standardized protocols (non-central). Oral test based on case resolution. 120 min for test [44]
6. Discussion of protocols and procedures	▪ Oral and/or written discussion of protocols and procedures [38]
7. Not specified written test	▪ Centrally standardized national written test. 120 min for test [44]
Practical test	
Types of tools	Description of practical test (specific for each study of the scoping review)
1. Simulated clinical case: objective structured clinical examination (*OSCE*) 2. Bedside test or test on the bed	▪ The exam stations are based on the stages of the nursing process [30,38] ▪ 3 h bedside test: I. Assessment of needs and problems, analyses and planning II. implementation and evaluation of nursing activities III. reflections and final judgment [31] ▪ Students take care of a patient in need of comprehensive medical and nursing care for 3 h [21] ▪ The test is performed after the written examination and lasts 4 h [36] ▪ The test is carried out after the written test, but the duration and method of carrying out the test are not specified [38] ▪ 120 min for each test on one patient [44]
3. Decontextualized practical test	▪ Test that focuses exclusively on the evaluation of the student’s technical competencies not contextualized in a clinical case. For example, material preparation for the measurement of Central Venous Pressure [38]
Not practical test	[29,32,33,34,35,37,39,40,41,42,43,45,46,47,48,49,50]

Legend: CAT = computerized adaptive testing; MCQ = multiple-choice question; MEQ = modified essay questions; NCJMM = National Clinical Judgment Measurement Model; NGN = Next-Generation NCLEX; OSCE = objective structured clinical examination; SCCRB = Standard Clinical Competencies Record Book.

## Data Availability

Data is contained within the article or Supplementary Materials.

## Supplementary Materials

The following supporting information can be downloaded at https://www.mdpi.com/article/10.3390/nursrep15080299/s1: Table S1: PRISMA_2020_checklis [27].

## Public Involvement Statement

No public involvement in any aspect of this research.

## Guidelines and Standards Statement

This manuscript was drafted against The PRISMA 2020 statement: an updated guideline for reporting systematic reviews (See Table S1 in the Supplementary Materials).

## Abbreviations

The following abbreviations are used in this manuscript:

AACN	American Association of Colleges of Nursing
ADN	Associate Degree in Nursing
AIDS	Acquired Immune Deficiency Syndrome
AINEC	Association of Indonesian Nurse Education Centre
AJCCN	ASEAN Joint Coordinating Committee on Nursing
ASEAN	Association of Southeast Asian Nations
ATI	Assessment Technologies Institute
BSN	Bachelor of Science in Nursing
CASN	Canadian Association of Schools of Nursing
CAT	Computerized Adaptive Testing
CEBN/ECBSI	Canadian Examination for Baccalaureate Nurses/l’Examen Canadien du Baccalaureate en Sciences Infirmières
CRNE	Canadian Registered Nursing Exam
CGPA	Cumulative Grade Point Average
COR	Conservation of Resources
EFN	European Federation of Nurses Associations
ENC	Eswatini Nursing Council
ETP	Entry to Practice Competencies
ETPC	Entry to Practice Competencies Client-Centered
GSE	General Self-Efficacy
HIV	Human Immunodeficiency Virus
INCE	Indonesian Nursing Competency Examination
JD-R	Job Demand–Resources
KNLE	Korean Nursing Licensing Examination
MCQs	Multiple-choice question
MEQ	Modified Essay Questions
NCFE	Swedish National Clinical Final Examination
NCLEX	National Council Licensure Examination
NCLEX-RN	National Council Licensure Examination for Registered Nurses
NCSBN	National Council of State Boards of Nursing
NCJMM	National Clinical Judgment Measurement Model
NGN	Next-Generation NCLEX
NL	Newfoundland and Labrador
NLE	Nursing Licensing Exam
NMC	Nursing and Midwifery Council
NMC-LE	Nursing and Midwifery Council- Licensure Examination
OSCEs	Objective Structured Clinical Examination
PLD	Performance Level Description
QSEN	Quality and Safety Education for Nurses
RN	Registered Nurse
SCCRB	Standard Clinical Competencies Record Book
SNLE	Saudi Nursing Licensure Examination
TB	Tuberculosis
TEAS	Test of Essential Academic Skills
USA	United States of America

## Appendix A

### Appendix A.1. Search Strategy (PubMed)

Search	Query	Results
#9	Search: (((((nursing student*[Title/Abstract]) AND (undergraduate or bachelor or university)) OR (“Students, Nursing”[Mesh])) AND (“final exam*”[Title/Abstract] OR “licensure exam*”[Title/Abstract] OR licensure*[Title/Abstract])) OR (“Licensure, Nursing”[Mesh])) NOT (pre-licensure[Title/Abstract]) Filters: Abstract, English, from 2000/1/1–2024/12/1	989
#8	Search: #6 + #7 (((((nursing student*[Title/Abstract]) AND (undergraduate or bachelor or university)) OR (“Students, Nursing”[Mesh])) AND (“final exam*”[Title/Abstract] OR “licensure exam*”[Title/Abstract] OR licensure*[Title/Abstract])) OR (“Licensure, Nursing”[Mesh])) NOT (pre-licensure[Title/Abstract])	4809
#7	Search: pre-licensure [Title/Abstract]	455
#6	Search: #3 + #2 + #1 + #5 + #4 ((((nursing student*[Title/Abstract]) AND (undergraduate or bachelor or university)) OR (“Students, Nursing”[Mesh])) AND (“final exam*”[Title/Abstract] OR “licensure exam*”[Title/Abstract] OR licensure*[Title/Abstract])) OR (“Licensure, Nursing”[Mesh])	4991
#5	Search: “final exam*”[Title/Abstract] OR “licensure exam*”[Title/Abstract] OR licensure*[Title/Abstract]	9223
#4	Search: “Licensure, Nursing”[Mesh]	4670
#3	Search: nursing student*[Title/Abstract]	20,684
#2	Search: undergraduate OR bachelor OR university	16,184,164
#1	Search: “Students, Nursing”[Mesh]	30,382

### Appendix A.2. Data Extraction Instrument

Author (s)	
Year of publication	
Country	
Study design	
Population	
Aim	
KEY FINDINGS	
Which competency framework is assessed during the nursing licensure?	
What are the expected performance levels?	
What types of tools are used for competencies assessment?	
